# Medication Errors Associated With Adverse Drug Reactions in Iran (2015-2017): A P-Method Approach

**DOI:** 10.15171/ijhpm.2018.91

**Published:** 2018-09-18

**Authors:** Zahra Karimian, Mehrnaz Kheirandish, Naghmeh Javidnikou, Gholamreza Asghari, Fariba Ahmadizar, Rassoul Dinarvand

**Affiliations:** ^1^Department of Assessment and Control on Prescribing and Use of Medicines and Health-Related Products, Iran Food and Drug Administration, Ministry of Health and Medical Education, Tehran, Iran.; ^2^Department of Pediatrics, Iran University of Medical Sciences, Tehran, Iran.; ^3^Department of Epidemiology, Erasmus University Medical Center, Rotterdam, The Netherlands.; ^4^Department of Pharmaceutics, School of Pharmacy, Tehran University of Medical Sciences, Tehran, Iran.

**Keywords:** Medication Errors, Preventable Adverse Drug Reactions, P-Method, Preventability, Pharmacovigilance, Patient Safety

## Abstract

Medication errors are the second most common cause of adverse patient safety incidents and the single most common preventable cause of adverse events in medical practice. Given the high human fatalities and financial burden of medication errors for healthcare systems worldwide, reducing their occurrence is a global priority. Therefore, appropriate policies to reduce medication errors, using national data and valid statistics are required. The primary objective of this study was to provide a national ‘characteristic profile’ of medication error-associated adverse drug reactions (ADRs), which are also known as preventable ADRs (pADRs). A retrospective study of pADR reports submitted to the national pharmacovigilance center (PCV) within Iran’s Food and Drug Administration was conducted over a 2-year period (2015-2017). Preventability Method (P-Method), which is a standardized tool developed and recommended by the World Health Organization (WHO), was used for preventability assessment. The results of the analyses revealed that while the number of pADRs increased from year one to two (601 to 630), their proportion out of all ADRs per year decreased (7.32% to 6.44%). The percentage of pADRs was higher in females (61.01%) and adults (83.27%), and the highest number of reports were received by nurses (71.57%). Having ‘a documented hypersensitivity to an administered drug or drug class’ was the most common preventable factor in both years (61.23% and 54.29% respectively), and ‘anti-infectives used systemically’ were the medication class which primarily contributed to both serious (53.29%) and non-serious pADRs (39.19%). The specific characteristics of medication errors associated with ADRs from this study, especially the preventable criteria which led to their occurrence, can help devise more specific preventative policies.

## Background

Medication errors are defined as an “unintended failure in the treatment process that lead to, or have the potential to lead to, patient harm.”^1^ They are classified via different approaches, such as *psychological mechanisms*,^2^ their *level of severity*,^3,4^ or the *processing stage* in which they occur.^5,6^ Regardless of the system employed for their classification, “medication errors are the single most common preventable cause of adverse events in medical practice, and a major public-health burden with an estimated annual cost of US$42 billion, almost 1% of global expenditure on health.”^7,8^ One estimate by the US Institute of Medicine reported that medication errors cause 1/131 outpatient and 1/854 inpatient deaths.^9^ Moreover, according to a report by the Iran’s Ministry of Health and Medical Education (MoHME), annual costs for prolonged hospitalization and extra care due to medication errors exceeded billions of Tomans (Iran’s currency).^10^

Given the high human fatalities and financial cost of medication errors, the World Health Organization (WHO) launched its third Global Patient Safety Challenge – *Medication Without Harm* – in March 2017 and announced that one of its major goals is to reduce the level of severe, avoidable harm related to medications by 50% over 5 years, globally.^11^

To date, a number of meta-analysis and review studies have been carried out on medication errors in Iran.^12,13^ However, the data from the individually reviewed studies had not used the same standardized definitions or detection methods for medication errors, or the focus was on its incidence within specific classes of drugs, healthcare professions or clinical settings.

According to the guidelines for good pharmacovigilance practice by the European Medicines Agency (EMA), as well as the definitions and standards in the E2A guidelines of the International Conference on Harmonisation (ICH), an adverse drug reaction (ADR) is defined as “a response to a medicinal product which is noxious (harmful) and unintended when a causal relationship between a drug and an adverse occurrence is suspected. ADRs may arise from the use of a product within or outside the terms of the marketing authorization, including off-label use, overdose, misuse, abuse and medication errors.”^14,15^ Furthermore, according to the National Coordinating Council for Medication Error Reporting and Prevention (NCC MERP), not all ADRs are caused by an error, however, the use of a drug as a result of an error is considered to be preventable.^16^ Therefore, medication errors associated with ADRs are also known as preventable ADRs (pADR). Since medication errors are a leading cause of pADRs, and national pharmacovigilance centers (PVCs) are considered to be a reliable source of ADR detection,^17-19^ the evaluation of pADRs appeared to be a logical first step in characterizing a subset of medication errors in Iran which could in turn inform potential interventions to reduce their occurrence.^20^

Since national statistics and comprehensive data regarding medication errors continue to be limited, this study aimed to develop a characteristic profile of medication errors using data from a regulatory body which are consistently collected, assessed and utilized in evidence-based policy making.

## Methods

### Definitions

The definitions and explanations for the terms and variables in this study are provided below:

*ADR –* Any noxious and unintended response to a drug which occurs at doses normally used in humans for prophylaxis, diagnosis, or therapy of disease, or for the modifications of physiological function, where a causal relationship between the drug and an adverse event is suspected. The ICH guidelines note that ADRs may arise from the use of medications within or outside the marketing authorization terms, which includes off-label use, overdose, misuse, abuse, and medication errors.^15^

*pADR* – A pADR is harm caused from drug use following a medication error, in which case the Preventability Method (P-Method) can be applied to determine the preventable criteria which led to its occurrence.^16^

*Serious ADR* ***– ***According to the ICH E2A criteria, serious ADRs are defined as reactions to a drug that result in death, permanent injury, significant disability/incapacity, or birth defect, and those requiring hospitalization or prolongation of existing hospitalization.^15^

*Medication classification* – Medications were classified according to the WHO’s Anatomical Therapeutic Chemical (ATC) classification system, the active substances are divided into different groups according to the organ or system on which they act and their therapeutic, pharmacological and chemical properties. Drugs are classified in groups at five different levels. For the purposes of this study, data classification was analyzed at the first level which divides medicines in to 14 main groups.^21^

*Patient age groups* ***– ***The age classification referenced in this study is that used by national health organizations to reimburse patients for medical expenses, which is also listed in the patient criteria for admission to pediatric (0–17 years old) and adult (≥18 years old) healthcare facilities.

### Data Source/Data Collection

The data for this study was obtained through the national PVC within Iran’s Food and Drug Administration (FDA). The Iranian PVC became a full-member of the International Drug Monitoring Program of the WHO since 1998 and has been submitting ADR reports to the Uppsala Monitoring Centre since then. In 2013, the Moroccan PVC pioneered a multicenter study which evaluated the P-Method as an instrument for detecting and assessing pADRs, in which Iran was among a select number of participating countries.^22^ In 2015, the Iranian PVC officially adopted P-Method to assess ADR preventability, and later incorporated certain modifications to this method, adjusting for national/domestic data. The PVC receives ADR reports documented in Yellow Cards (paper-based and online forms), which are standardized forms used to collect information about a suspected adverse event for further analysis. The form includes information pertaining to the patient, adverse event, suspected drug(s), seriousness and outcome of the event, actions taken, and the reporter. This information is communicated through several sources to the PVC: patients and their caregivers, trained ADR representatives in healthcare facilities (hospitals and clinics), as well as the Drug Poison Information Center (DPIC) which forwards suspected ADR incidents from the general public. In this retrospective study, the PVC server was used to extract pADR reports for a 2-year period since the implementation of P-Method (Year 1: March 2015–March 2016, and Year 2: March 2016–March 2017).

### Data Assessment

All assessment methods and processes carried out at the Iranian PVC follow the guidelines and recommendations of the WHO. ADR reports received by the PVC first undergo ‘causality assessment’ using the WHO-UMC Causality Assessment System.^23^ This is a process used for establishing a causal relationship between a drug and an adverse event. Upon identification of an ADR, the next step is to decide whether it is preventable or non-preventable. pADRs can be assessed using P-Method; a validated tool that relies on explicit criteria for assessing preventability.^22^ This standardized tool aims to identify preventable risk factors (from 20 explicit criteria) that increase the probability of ADR occurrence in relation to three factors: healthcare professionals’ practices, patient behavior, and drug quality. The outcome of the preventability assessment using P-Method is one of the following: ‘preventable,’ ‘non-preventable,’ or ‘not assessable.’ The preventability criteria used in P-Method are presented in Table S1 (Supplementary file 1). If none of the preventability criteria are selected for an ADR, it is considered as ‘non-preventable.’ Answering ‘yes’ to any of the criteria indicates that the ADR is ‘preventable’ and has a known risk factor. In year 1, the list included 20 preventable criteria as outlined in the original P-Method, however, in year 2, the Iranian PVC added ‘wrong drug’ as a new criterion to the list (number 9). The addition of this category was decided when certain ADRs were determined as preventable, but their preventable cause did not exactly match or could not fit in to any of the existing preventable categories.

### Data Verification

To rule out any systematic error or bias, and to take in to account for variability due to the subjective nature of applying P-Method, a random sample including 10% of all the pADR reports in each year was selected and re-assessed for preventability by an independent evaluator at the FDA. This was done as part of an internal audit system to provide additional assurance of our data prior to being used by decision-makers at the FDA for developing national preventative policies. The primary application of P-Method was carried out by two experts at the PVC (NJN and MK). Photocopies of the original Yellow Cards (reports) for the randomly selected samples were prepared and the confidential patient information was redacted prior to being submitted to the FDA. Preventability assessment was repeated for each ADR by a clinical pharmacist at the FDA (ZFK), and the results were compared to determine the percentage of agreement between the reviewers. If the inter-reviewer agreement for the random pADR sample was >95%, it did not warrant further reassessment by additional experts.

### Statistical Analysis

Descriptive statistics were used to summarize the rates of pADRs (frequency of pADRs among all reported ADRs in 2 years) by patient characteristics and event seriousness. The annual prevalence rates of pADRs were calculated and their preventable categories were reported for years 1 and 2. Further analysis was performed for the medication classes involved and reporters of the incidents (nurse, pharmacist, general practitioner, specialist, patient and other) over the two-year period. Since the preventability assessment tool (P-Method) was modified from year 1 to 2, the rates and preventable criteria for pADRs were reported separately for each year, but not compared statistically. All other factors such as patient characteristics, pADR seriousness, medication class, and the reporters of the incidents were reported for both years combined. Between groups, qualitative data were compared using 95% CI for rates reported.******The analyses were performed using Microsoft Excel software (v15.0, 2013) and SPSS version 18.0 (IBM Corp., Armonk, NY, USA).

## Results

### Prevalence Rates

From a total of 17 988 ADR reports received by the PVC over 2 years, 1231 cases were identified as pADRs (6.84%) which were included in this study. In year 1, of a total of 8205 ADR reports, 601 (7.32%) were preventable, while in year 2, of 9783 ADR cases, 630 (6.44%) were identified as preventable.

### Preventability

Data verification revealed a 98.4% inter-reviewer agreement in the preventability assessment of ADRs, which did not warrant further assessment by additional experts. The most frequent preventable risk factor leading to pADRs, in both years was ‘having a documented hypersensitivity to an administered drug or drug class’ (61.23% and 54.29% respectively). The five most prevalent and least frequent preventable criteria for pADRs are compared in Table 1. Complete results are provided in Supplementary file 1.

**Table 1:** The 5 Most and Least Common Preventable Criteria for pADR Occurrence Compared Between Years 1 and 2

**Preventability Criteria**	**Year 1 (20 Criteria)**	**% pADRs/** **All pADRs**	**Year 2 (21 Criteria)**	**% pADRs/All pADRs**
**Most prevalent**	1. Having a documented hypersensitivity to an administered drug or drug class	61.23%	1. Having a documented hypersensitivity to an administered drug or drug class	54.29%
	2. Inappropriate prescription for patient’s clinical condition	6.99%	2. Wrong drug	20.01%
	3. Inappropriate prescription according to patient characteristics	5.16%	3. Drug administration error	6.67%
	4. Drug administration error	4.66%	4. Poor quality drug administered	5.56%
	5. Poor quality drug administered	3.83%	5. Wrong indication	3.65%
**Least prevalent**	16. Withdrawal syndrome due to abrupt discontinuation of drug	0.83%	17. Necessary medication not given	0.32%
	17. Expired drug administered	0.67%	18. Withdrawal syndrome due to abrupt discontinuation of drug	0.16%
	18. Therapeutic duplication	0.63%	19. Expired drug administered	<0.10%
	19. Incorrect drug dosage formulation administered	0.48%	20. Incorrect laboratory or clinical monitoring of medicine	<0.10%
	20. Non-compliance	0.16%	21. Counterfeit drug administered	<0.10%

Abbreviation: pADRs, preventable adverse drug reactions.

The rates of pADRs in 2 years was stratified by gender, age, and seriousness which is presented in Table 2.

**Table 2:** The Prevalence Rates of pADRs by Gender, Age, and Seriousness

**Patient and pADR Characteristics**	**Year 1 and 2**	
	**Proportion out of Total pADRs (% pADR)**
Gender	
Female	751/1231 (61.01%)
Male	480/1231 (38.99%)
Age	
Pediatric (<18 y)	206/1231 (16.73%)
Adult (≥18 y)	1025/1231 (83.27%)
Seriousness	
Serious	74/1231 (6.01%)
Non-serious	1157/1231 (93.99%)

Abbreviation: pADRs, preventable adverse drug reactions.

### Medication Classification

The medication classes most commonly involved in pADRs over the 2-year period were systemic anti-infectives 53.29%, followed by nervous system 15.43%, antineoplastic and immunomodulating agents 8.94%, alimentary tract and metabolism 7.15%, and systemic hormonal preparations 4.87%. In both years, anti-infective agents as well as antineoplastic and immunomodulating agents were the most common causes of serious pADRs, 39.19% and 29.73% respectively (Table 3).

**Table 3:** Top 5 Medication Classes Most Commonly Involved in pADR Occurrence in 2 years

**ATC Codes**	**Top 5 Medication Classes Involved in pADR Occurrence**	**pADR/All ADRs** **No. (%)**	**Serious pADR /All Serious ADRs** **No. (%)**
J01, J05–J06	Anti-infectives for systemic use	656 (53.29)	29 (39.19)
N01, N02 & N03	Nervous system	190 (15.43)	5 (6.76)
L01, L03–L04	Antineoplastic and immunomodulating agents	110 (8.94)	22 (29.73)
A01–A09	Alimentary tract and metabolism	88 (7.15)	0 (0.0)
H01–H02	Systemic hormone preparations	60 (4.87)	6 (8.11)
-	Other medication classes	127 (10.32)	12 (16.22)
	Total Reports (N)	1231	74

Abbreviation: pADRs, preventable adverse drug reactions; ATC, Anatomical Therapeutic Chemical.

### Reporters

The majority of pADRs were reported by nurses 881 (71.57%), while the least number of reports among healthcare professionals was submitted by medical specialists (3.74%). Only one report was received from a patient, and the remaining were communicated by ‘others’ 25 (2.03%), which includes caregivers or other groups of healthcare providers. This information is illustrated in Figure .

**Figure:**
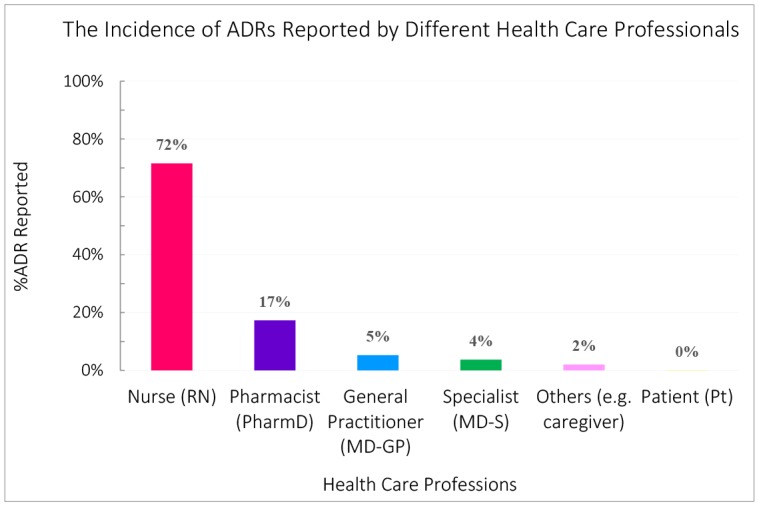
The Percentage of Preventable Adverse Drug Reactions (pADRs) Reports Submitted by Different Groups in 2 Years.

## Discussion

This is the first study to assess pADR data from the national PVC in Iran, using P-Method, since it was implemented in 2015.

The findings of this study showed that while the number of pADR cases increased from year one to two (601 to 630), their proportion out of all ADRs decreased (7.40% to 6.52%). This slight decline may reflect the impact of patient safety interventions by the MoHME. Nonetheless, there is continued need for work in this area. Furthermore, the rate of pADRs is generally reported to have a broad range, from 18.7%–80%,^22^ which depends on multiple factors such as each country’s health culture, resources, and healthcare system, clinical setting and patient population being studied. One of the main advantages of P-Method in comparison to other preventability assessment tools is that it reports definitive pADRs instead of all possible, probable and definitive cases. This could also explain the lower rates of pADRs found in this study. That said, the rate of pADRs in Iran from this study seems to be comparable to those reported by other countries such as Sweden and Morocco^24-26^ which are among the Collaborating Centers for the WHO’s International Drug Monitoring Program^27^ and had used P-Method for reporting national pADR rates.

Having ‘a documented hypersensitivity to an administered drug or drug class’ was the most common preventable factor leading to pADRs in both years. This is of particular interest since ‘anti-infectives used systemically’ were found to be the medication class that primarily contributed to the occurrence of both serious and non-serious pADRs. Given that antibiotics are among drug classes most commonly associated with hypersensitivity reactions and have a high per capita consumption in Iran,^28^ the importance of promoting the *rational use of medicines* and implementing more rigorous *antimicrobial stewardship programs*, in addition to ensuring *comprehensive medication history and reconciliation* could help prevent such ADRs. It should also be noted that this type of pADR could be a result of prescribing, dispensing and/or administration errors. Therefore, developing appropriate preventative measures and educational programs in the above-mentioned areas should be among the priorities of all healthcare professional groups.

This study found that the rate of pADRs was higher in females compared to males. This was similar to a study conducted by the Moroccan PVC where the authors reported that from 180 pADR cases over a period of 3 years, 53% occurred in women.^18^ The review by Aagaard and colleagues which assessed the global patterns of ADR over a decade (2000-2009) similarly reported higher rates of ADRs in females (60%). The difference in pADR prevalence rates between the sexes could be due to multiple factors, such as difference in gender-associated healthcare-seeking behavior.^29^

Medication errors have been recognized as an area of concern in all age groups. As with other healthcare–related adverse events, patients at the extremes of age (pediatrics and geriatrics) are most vulnerable. Furthermore, due to inadequate clinical trials for medication use in pediatrics, children are at higher risk of developing unknown or rare ADRs. Nevertheless, this study showed that compared to adults, the rate of pADRs was considerably lower in pediatrics. This possibly reflects additional safety check in medication prescribing and administration by healthcare professionals in this particular patient population. The lower prevalence of chronic disorders and lighter medication burden among children, as well as their limited communication skills are other possible reasons.

Apart from ‘anti-infectives used systemically,’ the results showed that similar to high-income countries, ‘antineoplastic agents’ were another major contributor to pADR occurrence.^30^ The National Drug Policy (developed by the Iran FDA) aims to ensure availability, accessibility and affordability of medications, including those used for cancer treatment. Therefore, monitoring the impact of implementing national clinical guidelines will be beneficial for the safe use of these high-risk medication classes.

The knowledge, attitude, and perception of various healthcare professionals regarding pharmacovigilance, as well as the reasons hindering their reporting of ADRs have been studied extensively.^31,32^ The percent of ADRs reported by different healthcare professionals varies among countries and practice settings. There are over 1140 trained ADR representatives in Iran, the majority of which include nurses and pharmacists (>95%). In this study, the largest portion of pADR reports were received from nurses (Figure). The surveys directed by the Iranian PVC following annual training sessions indicate that each professional group can better teach colleagues in their own profession, it would be important to train more physicians in pharmacovigilance so that they can support each other to recognize and report ADRs, while an interdisciplinary approach where pharmacists assist other clinicians would also be useful.

Since its implementation in 2015, the application of P-Method in Iran has provided valuable insights. Analysis of pADRs in the first year demonstrated that certain pADRs could not be assigned to any of the existing preventability criteria. This resulted in the addition of a new criterion termed ‘wrong drug’ to the original P-Method list the following year. A retrospective sub-analysis of the pADRs assigned to this group revealed that the underlying preventable risk factors were primarily due to inadequate safety measures in the labelling and packaging of look-alike, sound-alike (LASA) drugs. In fact, the existence of confusing drug names has been determined as one of the most common causes of medication errors in Iran.^33^ Other risk factors leading to pADRs in this group included healthcare staff fatigue, physician’s illegible handwriting, and inappropriate work environment. Of note, the second most preventable criterion leading to pADRs in year two belonged to this newly added category. This may be due to this criterion encompassing a broader spectrum of risk factors, which did not require a more detailed analysis of each pADR report to determine whether it actually belonged to a more specific, existing category from the original list. To address this matter, a solution was proposed and recently adopted as policy by the FDA, which entailed further subdivision of this broad category into the more specific criteria noted above for future assessments of pADR.

## Limitations

The main limitation of this study is that since the preventability assessment tool (P-Method) was modified in the second year, the data could not be statistically compared between the two years to detect significant difference. Finally, since many ADRs could go unreported, the estimated pADR rate in this study is likely to be an underestimate of the true extent of ADR prevalence in Iran.

## Conclusion

The results of this study demonstrated that similar to several other countries, anti-infective and antineoplastic agents are major contributors to pADR prevalence in Iran.

Given the differences in healthcare systems, certain preventable criteria are expected to be specific to each country’s setting. Therefore, it is recommended that each country consider these differences when adopting standardized assessment tools such as P-Method for preventability assessment.

The specific preventable criteria predominantly causing pADRs revealed through this study will allow policy-makers to devise customized strategies for future prevention.

## Acknowledgements

The authors wish to express their sincere gratitude to Dr. Gloria Shalviri and Dr. Marjan Karimi for their contributions to the data used for this study.

## Ethical issues

Any private patient-related information on the ADR report forms were redacted for the purposes of this study, therefore, patient confidentiality guidelines were observed. No tests were conducted on human subjects for this research. Thus, it was exempt from seeking permission or authorization from an ethics committee.

## Competing interests

This research received a non-restricted grant from the Deputy for Science and Technology of the President’s Office. The contents of this article have not been influenced by the financial resource disclosed above and the authors declare that they have no conflicts of interest.

## Authors’ contributions

Conception and design: ZK and MK. Acquisition of data: NJ. Analysis and interpretation of data: ZK and MK. Statistical analysis: ZK and FA. Drafting of the manuscript: ZK. Critical revision of the manuscript for important intellectual content: MK, GA, and RD. Obtaining funding: ZK. Administrative, technical, and material support: MK. Supervision: MK.

## Authors’ affiliations

^1^Department of Assessment and Control on Prescribing and Use of Medicines and Health-Related Products, Iran Food and Drug Administration, Ministry of Health and Medical Education, Tehran, Iran. ^2^Department of Pediatrics, Iran University of Medical Sciences, Tehran, Iran. ^3^Department of Epidemiology, Erasmus University Medical Center, Rotterdam, The Netherlands. ^4^Department of Pharmaceutics, School of Pharmacy, Tehran University of Medical Sciences, Tehran, Iran.

## Supplementary files


Supplementary file 1 contains Table S1 and Complete list of preventable criteria for pADRs in years 1 and 2.
