# Supplementary material for: Medication Errors Associated With Adverse Drug Reactions in Iran (2015-2017): A P-Method Approach
Source: Int J Health Policy Manag. 2018 Sep 18;7(12):1090–6. doi: 10.15171/ijhpm.2018.91 (PMC6358654; doi:10.15171/ijhpm.2018.91)
Supplement: Supplementary file 1 — contains Table S1 and Complete list of preventable criteria for pADRs in years 1 and 2. [file ijhpm-7-1090-s001.pdf]

Table S1. P-Method Criteria (22) for 2 years

Related factors	Preventability Criteria	Yes	No	Unknown[1]	Not Applicable[2]
Healthcare professionals & practice	1. Incorrect dose?				
	2. Incorrect drug administration route?				
	3. Incorrect drug administration duration?				
	4. Incorrect drug dosage formulation administered?				
	5. Expired drug administered?				
	6. Incorrect storage of drug?				
	7. Drug administration error (timing, rate, frequency, technique, preparation, manipulation, mixing)?				
	8. Wrong indication?				
	9. Wrong drug?*				
	10. Inappropriate prescription according to characteristics of the patient (age, sex, pregnancy, other)?				
	11. Inappropriate prescription for patient's clinical condition (renal failure, hepatic failure, etc.), or another underlying pathology?				
	12. Documented hypersensitivity to administered drug or drug class?				
	13. Labelled drug-drug interaction?				
	14. Therapeutic duplication (prescription of 2 medicines or more with similar ingredient)?				
	15. Necessary medication not given?				
	16. Withdrawal Syndrome (due to abrupt discontinuation of treatment)?				
	17. Incorrect laboratory or clinical monitoring of medicine?				
Product / drug	18. Poor quality drug administered?				
	19. Counterfeit drug administered?				
Patient	20. Non-compliance?				
	21. Self-medication with non-over-the-counter (non-OTC) drugs?				

Abbreviation: P-Method: Preventability method

[1] A criterion that is not documented in the report form is considered unknown.

[2] A criterion is deemed ‘not applicable’ if it is not involved in ADR occurrence according to the reviewer evaluation.

* ‘Wrong drug’ is the preventable criterion added to the original P-Method list by the Iranian PVC

Complete list of preventable criteria for pADRs in years 1 and 2 separately:

Year 1 –

1. Having a documented hypersensitivity to an administered drug or drug class: 368 (61.23%)
2. Inappropriate prescription for patient’s clinical condition: 42 (6.99%)
3. Inappropriate prescription according to patient characteristics: 31 (5.16%)
4. Drug administration error (timing, rate, frequency, technique, preparation, manipulation, mixing)? 28 (4.66%)
5. Poor quality drug administered: 23 (3.83%)
6. Counterfeit drug administered: 14 (2.33%)
7. Incorrect storage of drug: 13 (2.16%)
8. Wrong indication: 11 (1.83%)
9. Labelled drug-drug interaction: 11 (1.83%)
10. Self-medication with non-OTC drug: 9 (1.50%)
11. Incorrect dose: 8 (1.33%)
12. Incorrect laboratory or clinical monitoring of medicine: 8 (1.33%)
13. Incorrect drug administration route: 7 (1.16%)
14. Necessary medication not given: 6 (1.00%)
15. Incorrect drug administration duration: 5 (0.83%)
16. Withdrawal Syndrome (due to abrupt discontinuation of treatment): 5 (0.83%)
17. Expired drug administered: 4 (0.67%)
18. Therapeutic duplication (prescription of 2 medicines or more with similar ingredient): 4 (0.67%)
19. Incorrect drug dosage formulation administered: 3 (0.5%)
20. Non-compliance: 1 (0.17%)

Total pADR cases = 601

Year 2 –

1. Having a documented hypersensitivity to an administered drug or drug class: 342 (54.29%)
2. Wrong drug: 126 (20%)
3. Drug administration error (timing, rate, frequency, technique, preparation, manipulation, mixing): 42 (6.67%)
4. Poor quality drug administered: 35 (5.56%)
5. Wong indication: 23 (3.65%)
6. Incorrect dose: 19 (3.02%)
7. Labelled drug-drug interaction: 7 (1.11%)
8. Incorrect drug administration duration: 6 (0.95%)
9. Incorrect drug administration route: 3 (0.48%)

10. Therapeutic duplication (prescription of 2 medicines or more with similar ingredient): 4 (0.63%)
11. Self-medication with non-OTC drug: 4 (0.63%)
12. Incorrect drug dosage formulation administered: 3 (0.48%)
13. Inappropriate prescription for patient's clinical condition (renal failure, hepatic failure…), or underlying pathology: 3 (0.48%)
14. Incorrect storage of drug: 2 (0.32%)
15. Inappropriate prescription according to characteristics of the patient (age, sex, pregnancy, other): 2 (0.32%)
16. Necessary medication not given: 2 (0.32%)
17. Withdrawal Syndrome (due to abrupt discontinuation of treatment): 1 (0.16%)
18. Non-compliance: 1 (0.16%)
19. Expired drug administered: 0 (0.00%) → Reported as <0.1%
20. Incorrect laboratory or clinical monitoring of medicine: 0 (0.00%) → Reported as <0.1%
21. Counterfeit drug administered: 0 (0.00%) → Reported as <0.1%

Total pADR cases = 630
